# High-resolution computed tomography and histopathological findings in hypersensitivity pneumonitis: a pictorial essay*

**DOI:** 10.1590/0100-3984.2014.0062

**Published:** 2016

**Authors:** Pedro Paulo Teixeira e Silva Torres, Marise Amaral Rebouças Moreira, Daniela Graner Schuwartz Tannus Silva, Roberta Rodrigues Monteiro da Gama, Denis Masashi Sugita, Maria Auxiliadora do Carmo Moreira

**Affiliations:** 1 MD, Radiologist, Volunteer at the Hospital das Clínicas da Universidade Federal de Goiás (UFG), Radiologist at Clínica Multimagem, Goiânia, GO, Brazil.; 2 PhD, Associate Professor in the Department of Pathology, Faculdade de Medicina da Universidade Federal de Goiás (UFG), Goiânia, GO, Brazil.; 3 MD, MSc, Pulmonologist at the Hospital das Clínicas da Universidade Federal de Goiás (UFG), Goiânia, GO, Brazil.; 4 MD, Radiologist, Graduate Student in Breast Imaging at Hospital do Câncer de Barretos, Barretos, SP, Brazil.; 5 MD, Pathologist, Assistant Professor at the Anápolis Unievangélica, Anápolis, GO, Brazil.; 6 PhD, Associate Professor at the Faculdade de Medicina da Universidade Federal de Goiás (UFG), Goiânia, GO, Brazil.

**Keywords:** Alveolitis, extrinsic allergic/pathology, Tomography, X-ray computed, Lung diseases, interstitial

## Abstract

Hypersensitivity pneumonitis is a diffuse interstitial and granulomatous lung disease caused by the inhalation of any one of a number of antigens. The objective of this study was to illustrate the spectrum of abnormalities in high-resolution computed tomography and histopathological findings related to hypersensitivity pneumonitis. We retrospectively evaluated patients who had been diagnosed with hypersensitivity pneumonitis (on the basis of clinical-radiological or clinical-radiological-pathological correlations) and had undergone lung biopsy. Hypersensitivity pneumonitis is clinically divided into acute, subacute, and chronic forms; high-resolution computed tomography findings correlate with the time of exposure; and the two occasionally overlap. In the subacute form, centrilobular micronodules, ground-glass opacities, and air trapping are characteristic high-resolution computed tomography findings, whereas histopathology shows lymphocytic inflammatory infiltrates, bronchiolitis, variable degrees of organizing pneumonia, and giant cells. In the chronic form, high-resolution computed tomography shows traction bronchiectasis, honeycombing, and lung fibrosis, the last also being seen in the biopsy sample. A definitive diagnosis of hypersensitivity pneumonitis can be made only through a multidisciplinary approach, by correlating clinical findings, exposure history, high-resolution computed tomography findings, and lung biopsy findings.

## Abstract (pt)

A pneumonite por hipersensibilidade é uma doença intersticial difusa, granulomatosa e de natureza inalatória, com inúmeros antígenos orgânicos e inorgânicos implicados. Nosso objetivo é ilustrar o espectro de anormalidades na tomografia de alta resolução do tórax e achados histopatológicos. Foram avaliadas, retrospectivamente, tomografias de alta resolução do tórax de pacientes com diagnóstico de pneumonite por hipersensibilidade estabelecido em correlação clínico-radiológica e clínico-radiológico-patológica em pacientes em que a biópsia esteve disponível. A pneumonite por hipersensibilidade é dividida, do ponto de vista clínico e didático, em aguda, subaguda e crônica, e as manifestações tomográficas da tomografia de alta resolução do tórax correlacionam-se com o tempo da evolução da doença, e por vezes se superpõem. Micronódulos centrolobulares, vidro fosco e aprisionamento aéreo são características tomográficas na manifestação subaguda, em que, histopatologicamente, se observam infiltrado inflamatório linfocítico, bronquiolite, graus variáveis de pneumonia em organização e células gigantes. Na fase crônica, a tomografia demonstra sinais de fibrose com bronquiolectasias de tração e eventualmente faveolamento, com predomínio superior, sendo observados também sinais de fibrose no estudo histopatológico. A abordagem multidisciplinar é definitiva no diagnóstico, que é realizado correlacionando-se as características clínicas, nexo causal de exposição, achados tomográficos e aspectos histopatológicos.

## INTRODUCTION

Hypersensitivity pneumonitis is a diffuse interstitial and granulomatous lung disease caused by the inhalation of any one of a large number of antigens^(1-3)^.

There are few epidemiological data on the disease. Studies focused on the form known as "farmer's lung" reported a prevalence of 2-12%; hypersensitivity pneumonitis can be highly prevalent in high-risk settings^(3)^. The microbial agents implicated in hypersensitivity pneumonitis include thermophilic actinomycetes that colonize decaying plant material, atypical mycobacteria found in hot tub water, and different fungi, including *Aspergillus, Candida*, and *Penicillium* species^(1,3)^. Another source is exposure to animal protein, including protein antigens from birds such as canaries, pigeons, and parakeets, with emphasis on the fact that contact can also occur from using bird feather pillows and blankets^(1)^. Lowmolecular-weight inorganic materials that lead to the formation of haptens, such as adhesives and paints containing isocyanates, can also cause the disease^(1)^. Identification of exposure can be quite difficult. In up to 40% of biopsy-confirmed cases, the causative agent is not identified^(2)^.

Although conflicting classification systems have been described in the literature, hypersensitivity pneumonitis is clinically classified as: acute-sudden onset, a few hours after contact; subacute-insidious exposure, with clinical symptoms lasting for a few weeks or up to four months after contact; and chronic-also by insidious exposure, the disease extending beyond four months and showing evidence of fibrosis in imaging and histopathological studies^(1-3)^. In its acute form, the disease is characterized by fever, myalgia, headache, cough, a feeling of chest tightness, and leukocytosis, usually by 4-12 hours after exposure. There are few data in the literature on tomographic and histopathological manifestations in this stage of the disease^(2,4)^. In the insidious forms (subacute and chronic) there is gradual onset of dyspnea, fatigue, cough with expectoration, anorexia and weight loss. After re-exposure to the antigen, there can be periods of exacerbation of clinical symptoms, which typically last 24 hours and tend to worsen over time^(1)^.

Experimental *in vivo* and *in vitro* studies have shown that nicotine has anti-inflammatory properties and exerts an inhibitory effect on the lymphocyte infiltrate in the lung, thus protecting smokers from developing hypersensitivity pneumonitis^(5,6)^. The protective effect of nicotine also explains the fact that the disease in this group of patients tends to be insidious, recurrent, and associated with a worse prognosis, since prevention in the acute inflammatory form would lead to a delayed diagnosis, with irreversible lung damage at the time of symptom onset^(5,6)^.

The diagnosis is based on the combination of the following: respiratory symptoms; absence of smoking; evidence of exposure or positivity for specific IgG antibodies on serological tests; suggestive findings on high-resolution computed tomography (HRCT) of the chest; > 30% lymphocytes in bronchoalveolar lavage fluid; and surgical lung biopsy findings, in cases of diagnostic uncertainty^(5)^. The clinical presentation is often nonspecific, and there are radiological and histopathological similarities with many other entities^(1,2)^.

## HIGH-RESOLUTION COMPUTED TOMOGRAPHY

The advent of computed tomography significantly improved the diagnostic accuracy of imaging methods, and abnormalities are now observed in more than 90% of patients with hypersensitivity pneumonitis^(1)^. The changes observed in HRCT are multiple and vary according to the stage of the disease (Table 1).

**Table 1:** Main tomographic and histopathological findings in hypersensitivity pneumonitis.

	Acute	Insidious manifestations	
		Subacute	Chronic
Tomographic findings	Ground-glass opacities and consolidations	Centrilobular ground-glass micronodules; sparse/diffuse ground-glass opacities; air trapping, usually lobular; cysts	Reticular opacities, in some cases peribronchovascular; honeycombing; ground-glass opacities; centrilobular micronodules and air trapping; predominance in the middle lung fields
Histopathological findings	Diffuse alveolar damage; inflammation; signs of vasculitis	Cellular bronchiolitis and chronic peribronchovascular lymphocytic and plasma cell inflammatory infiltrates; non-caseous granulomas, giant cells with cholesterol clefts; Schaumann bodies and foamy macrophages	Fibrosis with a pattern that resembles usual interstitial pneumonia or nonspecific interstitial pneumonia and a peribronchovascular distribution; Schaumann bodies, giant cells, and granulomas; architectural distortion

### Acute hypersensitivity pneumonitis

In acute hypersensitivity pneumonitis, the findings are characteristic of pulmonary edema, including large areas of ground-glass opacities, accompanied by areas of focal consolidation in some cases^(3)^. Given the rapid resolution of those features, tomography is rarely used in the evaluation of patients with the acute form of the disease^(2)^.

### Subacute hypersensitivity pneumonitis

**Figure 1:**
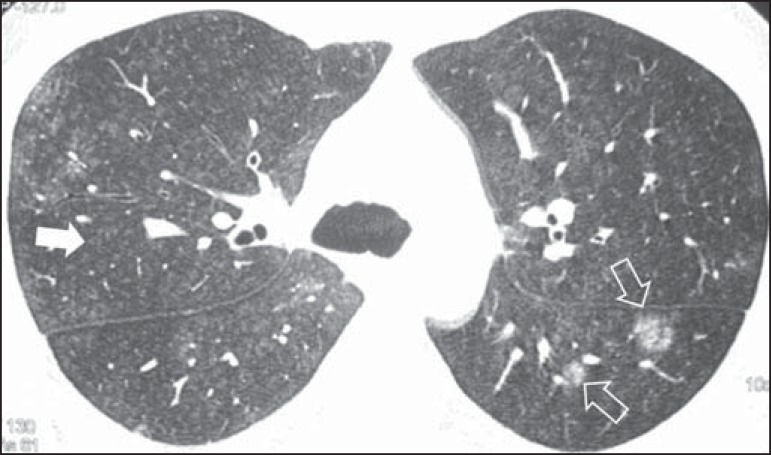
A 34-year-old male patient with a history of occupational exposure to wheat flour. Axial high-resolution computed tomography scan of the chest showing numerous centrilobular micronodules with ground-glass attenuation (solid arrow), as well as some larger ground-glass nodules in the upper segment of the left lower lobe (outlined arrows).

In subacute hypersensitivity pneumonitis, diffuse distribution of centrilobular ground-glass micronodules is a characteristic finding and may be the only or the predominant finding (Figure 1). The micronodules are usually numerous, with preferential distribution in the middle and lower lung fields^(1-3)^. Histopathology shows cellular bronchiolitis, peribronchiolar inflammation or organizing pneumonia^(1)^.

**Figure 2:**
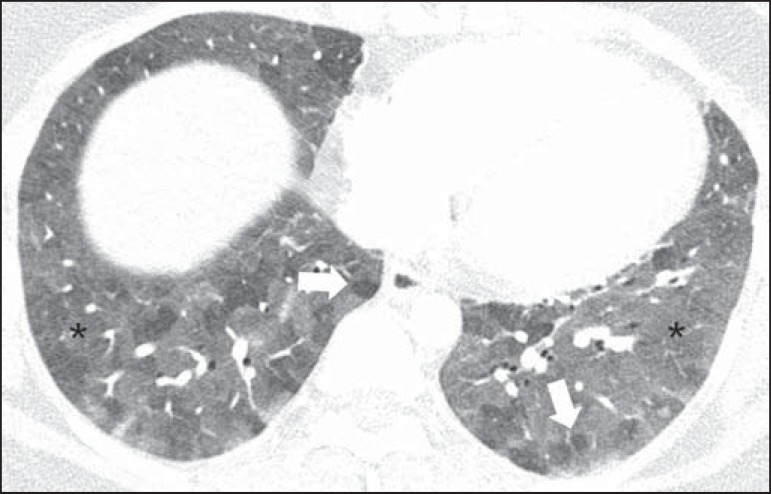
A 27-year-old female patient with a history of exposure to mold. Axial high-resolution computed tomography scans of the chest (lung window) at the level of the lower lobes showing extensive ground-glass opacities (asterisks), with overlapping foci of lobular air trapping (arrows).

Sparse or diffuse ground-glass opacities are often observed (Figure 2), with preferential distribution in the middle lung fields, and histopathology shows alveolar and interstitial inflammatory aspects, as well as organizing pneumonia or mild fibrosis^(1,3)^.

In up to 90% of patients, there is air trapping, usually of small extent; it is typically lobular and is best characterized in expiratory acquisition (Figure 2), probably due to cellular bronchiolitis or, less commonly, to constrictive bronchiolitis^(1,2,7)^.

Patients with subacute hypersensitivity pneumonitis can also present with the "head-cheese" sign, which is characterized by the combination of foci of ground-glass opacities, areas of normal attenuation, and air trapping, indicating infiltrative disease combined with bronchiolar obstruction^(1,3)^.

Small cysts are also observed in up to 13% of patients with the subacute form of the disease^(2)^.

### Chronic hypersensitivity pneumonitis

Distinguishing chronic hypersensitivity pneumonitis from other causes of fibrosis, such as usual interstitial pneumonia and nonspecific interstitial pneumonia, is important, since clinical management varies in each of these entities^(7)^.

In patients with chronic hypersensitivity pneumonitis, HRCT findings of a reticular pattern, traction bronchiectasis, and honeycombing are correlated with the finding of fibrosis in histopathological studies^(8)^. In the longitudinal axis, tomographic signs of fibrosis are described as prevalent in the middle lung fields, with relative sparing of the costophrenic sinuses and apexes^(1,3)^. Although infrequent, peribronchovascular predominance of the changes can be observed^(7)^.

**Figure 3:**
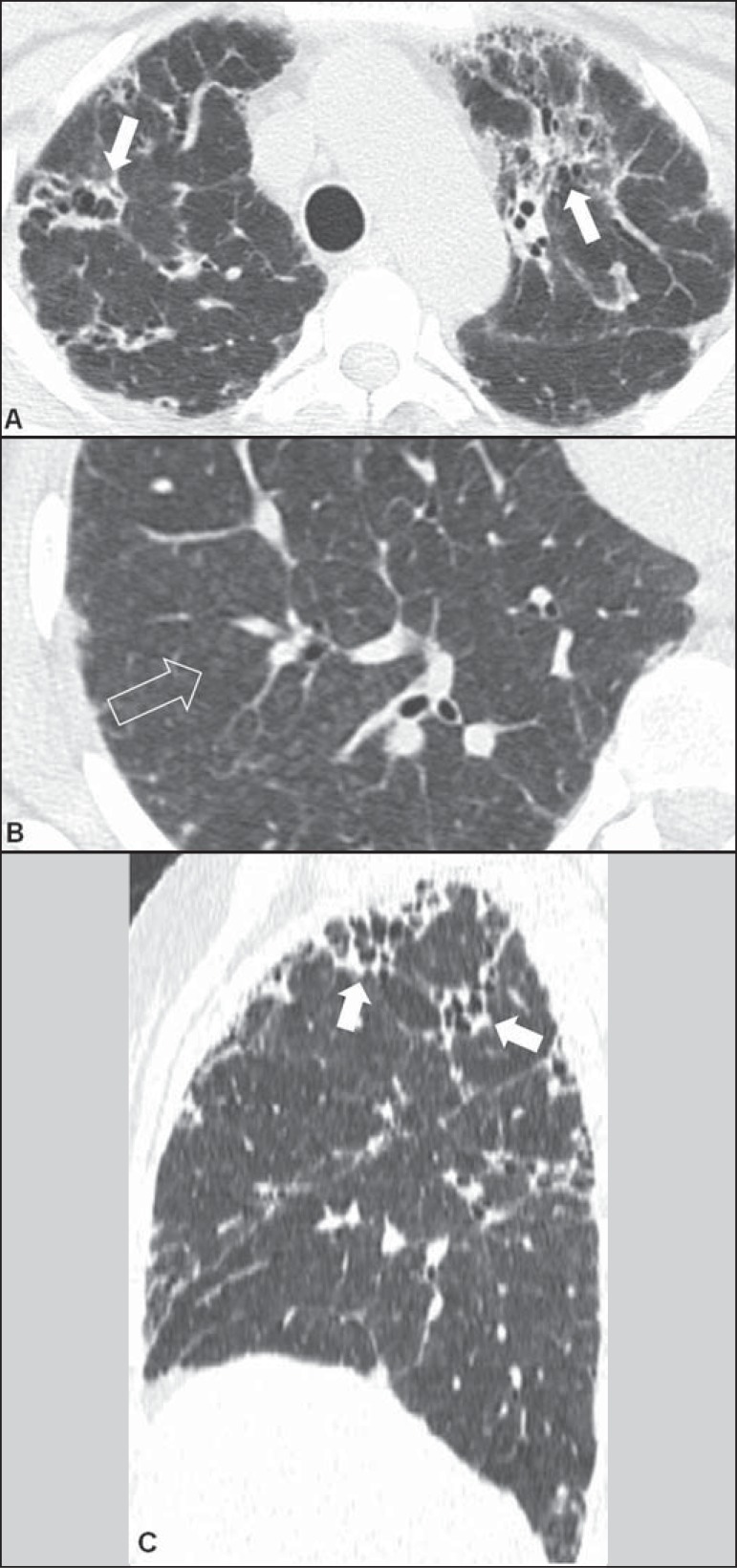
A 38-year-old female patient with a history of exposure to indoor mold. Axial high-resolution computed tomography scans of the chest (**A**,**B**) and the same images with sagittal reformatting (**C**). In **A**, peripheral reticular peribronchovascular opacities, with traction bronchiolectasis (arrows), showing a predominantly apical distribution, better identified in sagittal reformatting (**C**). In **B**, some associated centrilobular micronodules (arrow).

Associated centrilobular nodules, air trapping, ground-glass opacities dissociated from the areas of fibrosis, mild or no honeycombing, and relative preservation of the lung bases^(7)^ are characteristic tomographic findings in this stage, as shown in Figure 3.

## HISTOPATHOLOGICAL FINDINGS

### Acute hypersensitivity pneumonitis

Since lung biopsy is rarely performed in the acute form of the disease, there are few reports of histopathological presentation, which includes alveolar damage, with necrosis, inflammation, and vasculitis^(4)^.

### Subacute and chronic hypersensitivity pneumonitis

In subacute and chronic hypersensitivity pneumonitis, there are signs of cellular bronchiolitis, characterized by chronic peribronchovascular inflammation^(1,4)^. Non-caseating granulomas, also with peribronchovascular distribution, are observed, with or without giant cells^(1,4)^. In addition, there is evidence of chronic inflammatory interstitial disorder, the inflammatory cells primarily consisting of lymphocytes and plasma cells, as well as, to a lesser extent, eosinophils, neutrophils, and mast cells^(1,4)^.

**Figure 4:**
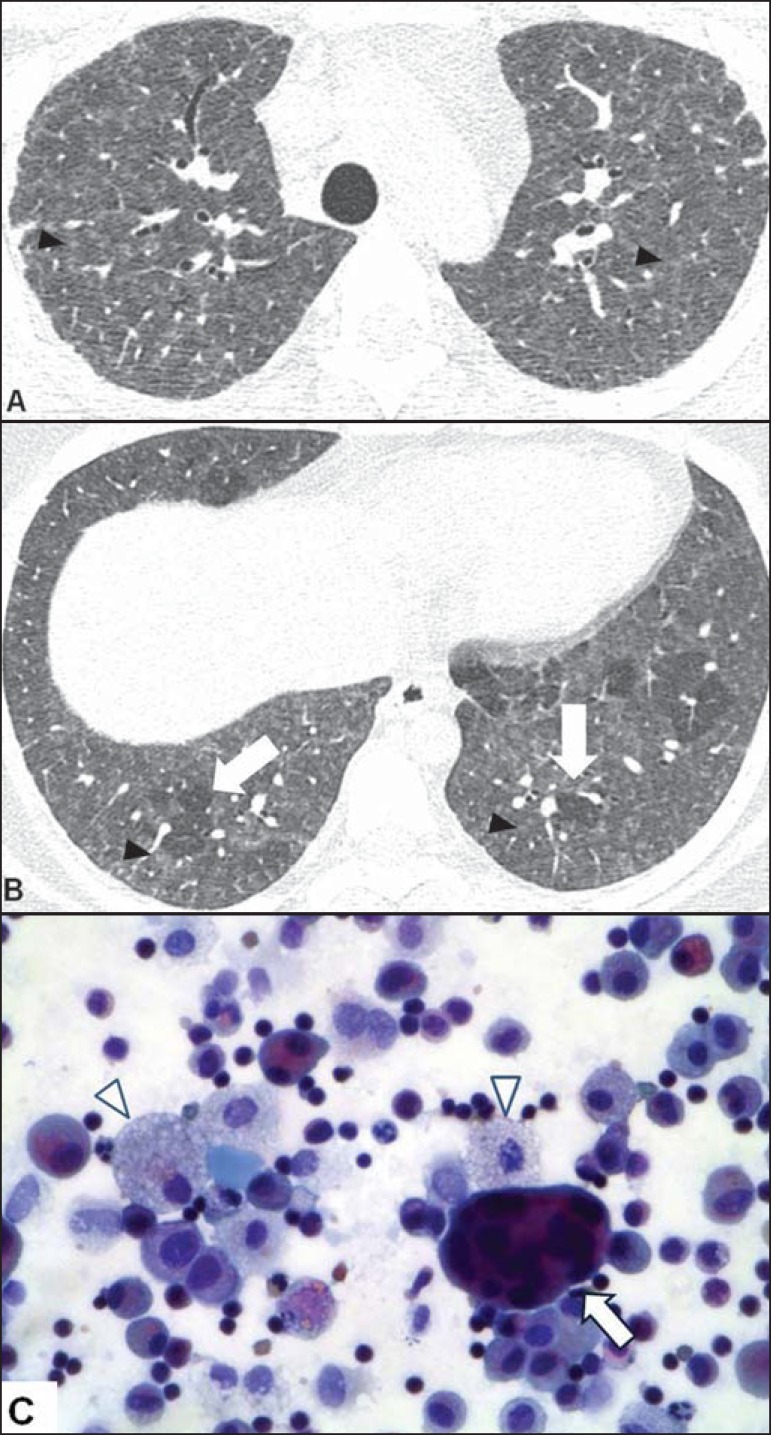
A 22-year-old female patient with a history of environmental exposure to ducks. **A,B**: Axial high-resolution computed tomography scans of the chest showing ground-glass opacities and centrilobular micronodules (arrowheads), in addition to incipient reticular opacities (in **A**), indicating initial fibrosis, and basal, in some cases lobular, air trapping (arrows in **B**). **C**: Papanicolaou-stained bronchoalveolar lavage fluid sample, showing a multinucleated giant cell (arrow), foamy histiocytes (arrowheads), and lymphocytes.

Giant alveolar and interstitial cells with cholesterol clefts, Schaumann bodies, or birefringent oxalate crystals are characteristic findings, and an accumulation of foamy macrophages can also be observed (Figure 4).

**Figure 5:**
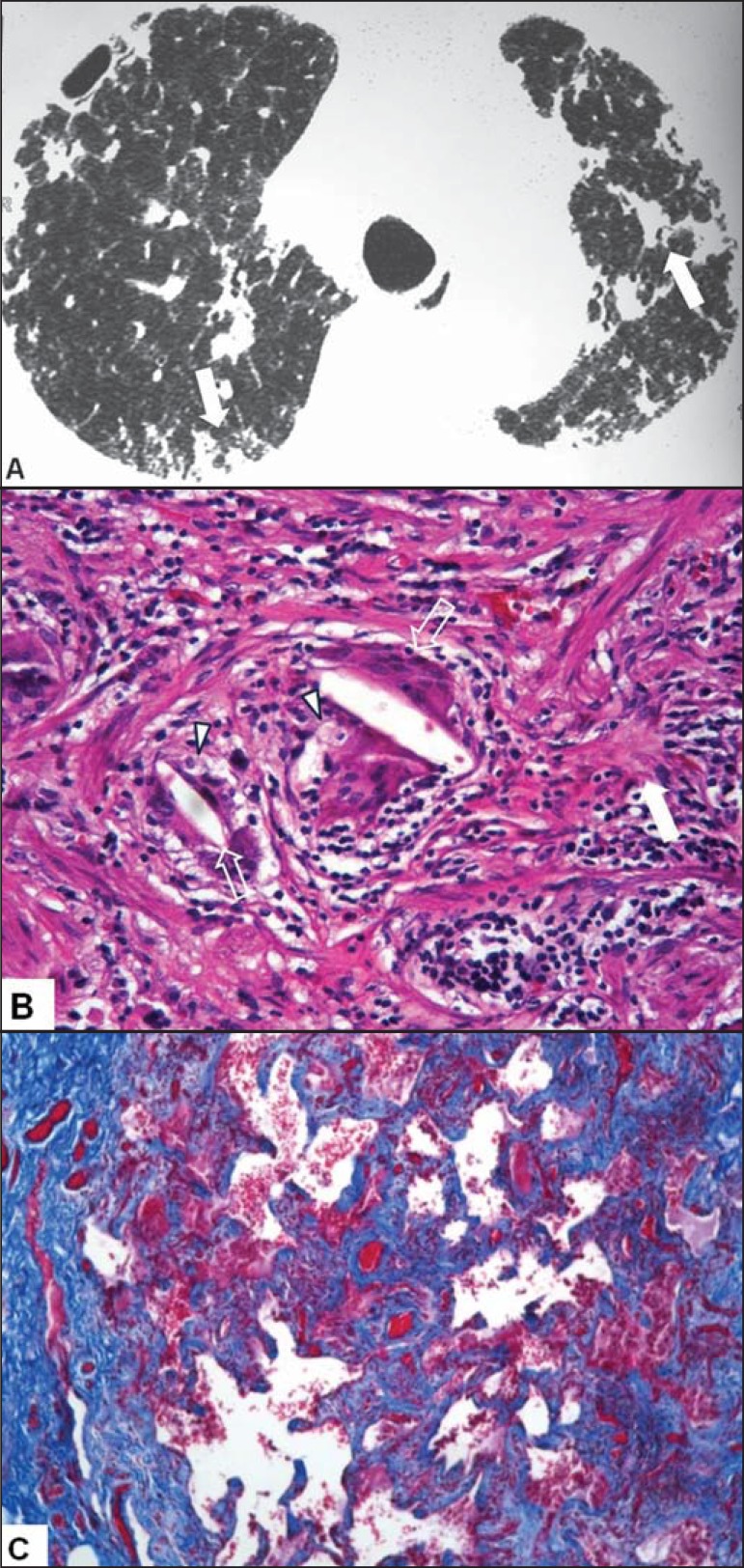
A 59-year-old female patient who bred pigeons and parakeets. **A:** Highresolution computed tomography scan of the chest showing architectural distortion, peripheral reticular opacities, and peribronchovascular opacities, accompanied by traction bronchiolectasis (arrows). **B:** Hematoxylin-eosin-stained lung biopsy sample, showing dense lymphocytic inflammation, foci of fibrosis (solid arrows), foamy histiocytes (arrowheads), and multinucleated giant cells with cholesterol crystals (outlined arrows). **C:** Foci of fibrosis highlighted in “blue” in Masson’s trichrome staining.

**Figure 6:**
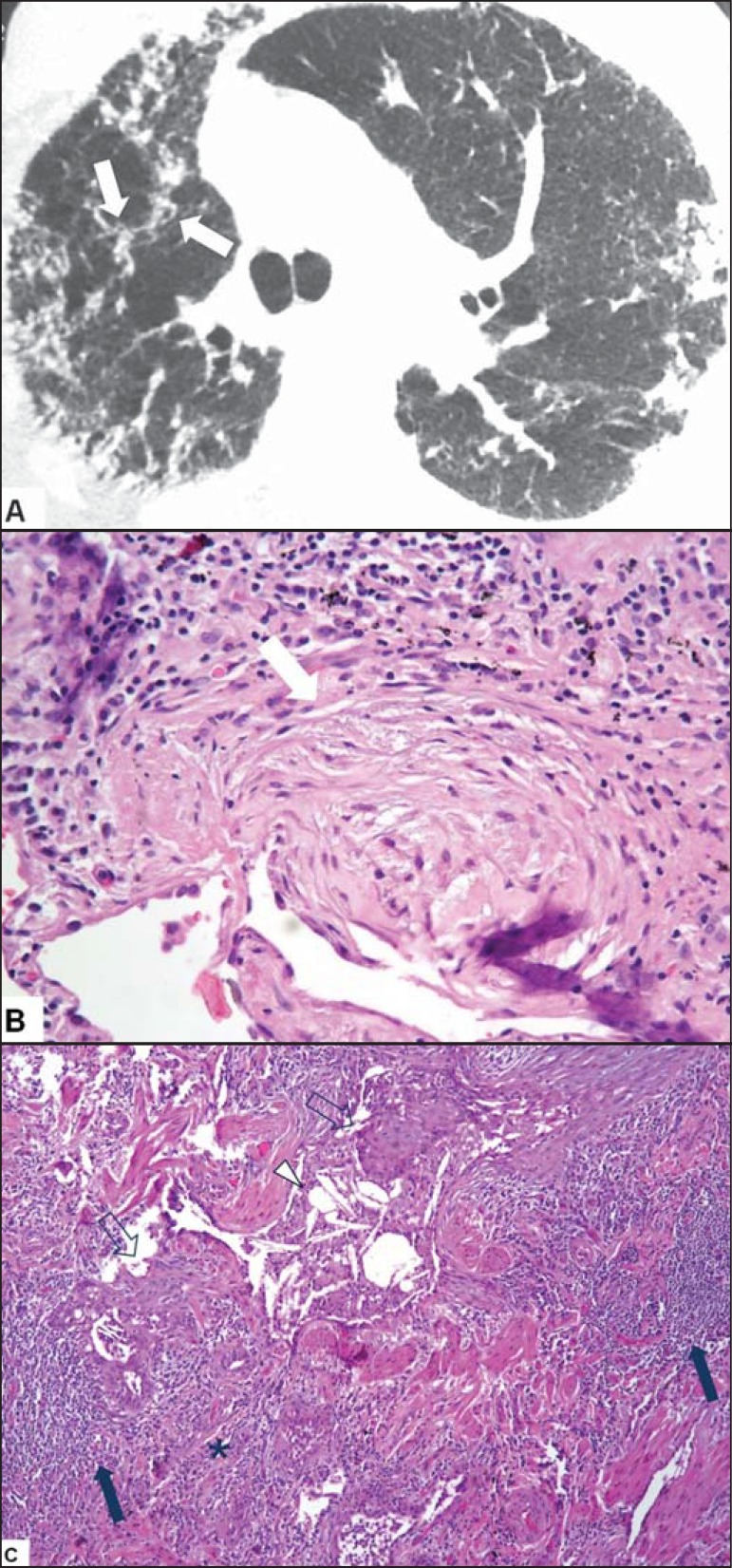
A 63-year-old female patient who worked in a corn husk storage facility. **A:** High-resolution computed tomography scan of the chest showing reticular opacities that were predominantly peripheral and some peribronchovascular foci in the right upper lobe (arrows). **B:** Histopathology of hematoxylin-eosin-stained surgical biopsy sample, showing areas of incipient fibrosis (arrow). **C:** Lymphocytic inflammation with lymphoid aggregates (solid arrows), foci of fibrosis (asterisk), giant cells with cholesterol crystals (arrowhead) and squamous metaplasia of the respiratory epithelium (outlined arrow).

The chronic form of the disease is characterized by fibrosis, which can be irregular and peribronchovascular (similar to that seen in usual interstitial pneumonia); subpleural, with architectural distortion and few inflammatory cells; or homogeneous, without architectural distortion (similar to that seen in nonspecific fibrotic interstitial pneumonia). In the last two situations, the presence of Schaumann bodies, giant cells, granulomas, and peribronchiolar fibrosis, as depicted in Figures 5 and 6, should lead to a diagnosis of hypersensitivity pneumonitis^(1)^.

## CONCLUSION

Hypersensitivity pneumonitis has multiple presentations in terms of the clinical symptoms, radiological manifestations, and histopathological aspects.

Ground-glass opacities, centrilobular ground-glass micronodules, and air trapping are characteristic of the subacute form of the disease. Those findings, together with fibrosis, characterize the chronic form of the disease^(2)^.

In the histopathological examination, signs of cellular bronchiolitis, chronic interstitial inflammation, granulomas, and giant cells are observed, as is fibrosis, in varying intensities^(1,4)^.

To ensure the reliable diagnosis of the disease, these findings should ideally be evaluated together, in a multidisciplinary context.
